# Plasmonic Titanium Nitride Nanohole Arrays for Refractometric Sensing

**DOI:** 10.1021/acsanm.3c03050

**Published:** 2023-11-14

**Authors:** Beyza Nur Günaydın, Mert Gülmez, Milad Torabfam, Zeki Semih Pehlivan, Atacan Tütüncüoğlu, Cemre Irmak Kayalan, Erhan Saatçioğlu, Mustafa Kemal Bayazıt, Meral Yüce, Hasan Kurt

**Affiliations:** † Faculty of Engineering and Natural Sciences, Sabanci University, Tuzla, Istanbul 34956, Turkey; ‡ SUNUM Nanotechnology Research and Application Centre, Sabanci University, Tuzla, Istanbul 34956, Turkey; § Department of Materials Science and Metallurgy, University of Cambridge, Cambridge CB2 3EQ, U.K.; ∥ Research Institute for Health Sciences and Technologies (SABITA), Istanbul Medipol University, Beykoz, Istanbul 34810, Turkey; ⊥ School of Engineering and Natural Sciences, Istanbul Medipol University, Beykoz, Istanbul 34810, Turkey; # Department of Bioengineering, Royal School of Mines, Imperial College London, London SW7 2AZ, U.K.

**Keywords:** transition metal nitrides, titanium nitride, plasmonics, nanohole array, refractometric sensing

## Abstract

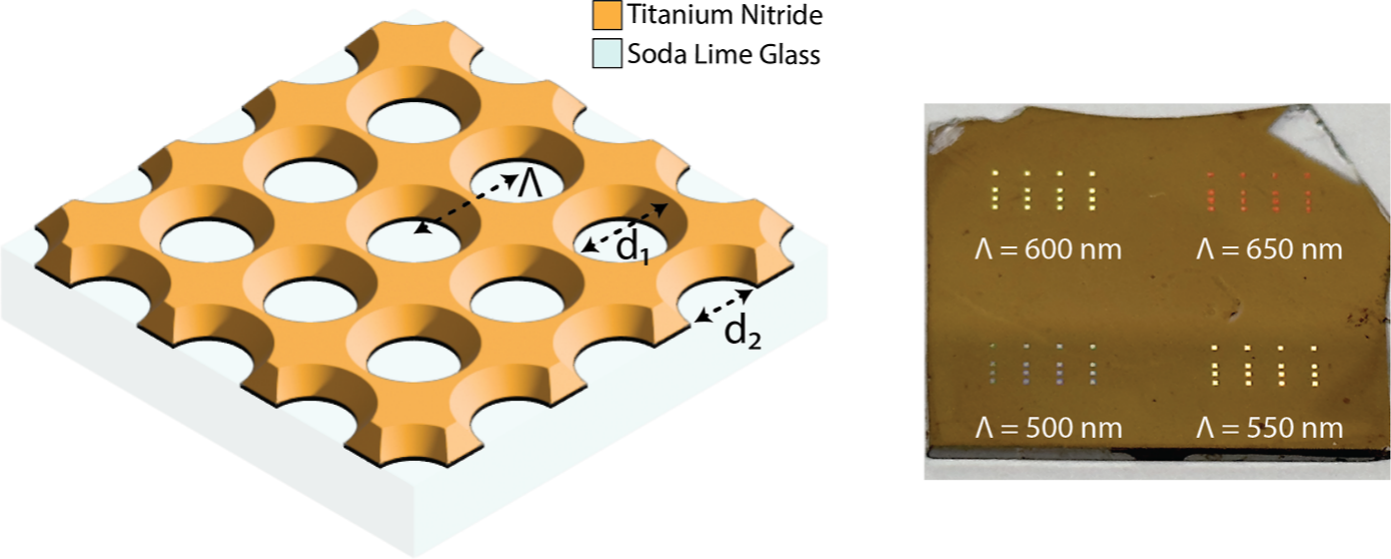

Group IVB metal nitrides have attracted great interest as alternative plasmonic materials. Among them, titanium nitride (TiN) stands out due to the ease of deposition and relative abundance of Ti compared to those of Zr and Hf metals. Even though they do not have Au or Ag-like plasmonic characteristics, they offer many advantages, from high mechanical stability to refractory behavior and complementary metal oxide semiconductor-compatible fabrication to tunable electrical/optical properties. In this study, we utilized reactive RF magnetron sputtering to deposit plasmonic TiN thin films. The flow rate and ratio of Ar/N_2_ and oxygen scavenging methods were optimized to improve the plasmonic performance of TiN thin films. The stoichiometry and structure of the TiN thin films were thoroughly investigated to assess the viability of the optimized operation procedures. To assess the plasmonic performance of TiN thin films, periodic nanohole arrays were perforated on TiN thin films by using electron beam lithography and reactive ion etching methods. The resulting TiN periodic nanohole array with varying periods was investigated by using a custom microspectroscopy setup for both reflection and transmission characteristics in various media to underline the efficacy of TiN for refractometric sensing.

## 1 Introduction

The field of plasmonics has had significant advances in the past 20 years, offering a bridge between the disciplines of optics and nanophotonics. Plasmonics comprises the interaction of light with surface plasmons, which are collective oscillations of free electrons in a sold at the nanoscale.^1^ Materials that display plasmon resonance—a characteristic that enables them to control and concentrate electromagnetic energy—are called plasmonic materials. The most widely used plasmonic materials are elemental gold and silver because of their high conductivity, small optical loss, and biocompatibility.^2,3^ Although these metals are utilized for numerous applications, some major drawbacks limit further utilization. Noble metals are not abundant and are thus expensive, constraining their large-scale implementations. The optical properties of plasmonic materials depend on the electron density. Given that gold exists in its elemental form, it is inherently unalterable, hence rendering its bulk dielectric characteristics unable to be modified by stoichiometry. The optical tunability of gold and silver nanoparticles can be modified by altering their particle size and geometry.^4^ The melting temperatures of bulk silver and gold are low and even lower when they are in the form of nanostructures, limiting their use in high-temperature applications. Plasmonic local heating events often cause thermal expansion, loss of mechanical rigidity, and shape distortion for nanostructures. Finally, the incompatibility with complementary metal oxide semiconductor (CMOS) fabrication methods is the most severe shortcoming of noble metals (i.e., gold) for plasmonic applications due to their propensity to function as a deep-level trap and recombination center. Specifically, charge carriers of opposing polarity tend to recombine at gold defects within silicon, resulting in their loss of current and causing contamination. Gold exhibits the formation of acceptor and donor levels inside the intrinsic energy band gap in silicon. The introduction of this substance into the semiconductor material results in its contamination and the subsequent formation of a deep-level trap.^2,4,5^ To remedy these fundamental issues, titanium nitride (TiN) has gained considerable attention as an alternative plasmonic material, offering advantages such as high-temperature stability, biocompatibility mechanical rigidity, chemical resistance, geopolitical availability, and compatibility with CMOS technology.^6–8^

The plasmonic materials provide opportunities for applications in numerous areas, such as nanochemistry, optical sensing, and light emission, thanks to their extraordinary absorption spectra, tunable optical properties, and ability to localize electromagnetic fields down to subwavelengths.^4,9^ Plasmonic materials improve the efficiency of photovoltaic devices,^10–12^ utilized through different geometries and techniques for biosensor applications,^13,14^ and provide large carrier density for boosting photocatalysis.^15,16^ Among these applications, refractometric sensing relies on detecting changes in the refractive index of various media, with applications in fields such as biosensing, environmental monitoring, and chemical analysis.^17^ Plasmonic materials have been widely employed in refractometric sensing due to their high sensitivity to local dielectric deviations.^17^

In general, transition metal nitrides (TMNs) are conductive ceramics that exhibit plasmonic characteristics (considerably high negative real part of the dielectric function in the visible and near-infrared spectrum). Group IVB, VB, and VIB metal nitrides are highly doped semiconductors, possess conductivity owing to free electrons from partially overlapping 3d and 2p orbitals, and exhibit plasmonic properties. Especially, group IVB metal nitrides (TiN, zirconium nitride, and hafnium nitride) exhibit a gold color and real part of (ε_1_) similar to the permittivity of Au with a higher imaginary part (ε_2_).^2,4^ The most intriguing property of TMNs is, in fact, highly tunable dielectric permittivity through metal/nitrogen stoichiometry.^4,18^ TMNs have remarkably high melting temperatures and show exceptional thermal stability; thus, TMN-based plasmonic devices are suitable for hot electron, photonic, and photothermal applications.^18–20^ These materials also show exceptional mechanical durability^2,18^ and chemical resistance to oxidation.^19^ TMNs display full-visible colors, highly reliant on the nanostructure’s physical shape.^21^ TMNs have compatibility with CMOS production and are used as conductive diffusion barriers for semiconductor devices.^18^ Although group IVB metal nitrides have strong plasmonic properties in the visible and NIR spectra regions, Ti is one of the cheapest and most abundant among group IVB metal nitrides. As a result, TiN was chosen for thin film fabrication and further plasmonic applications.

In this study, we demonstrate the tunable nature of the plasmonic properties of TiN thin films using reactive magnetron sputtering through the modulation of the Ar/N_2_ ratio. We also investigated the effects of oxygen contamination in TiN thin films using X-ray diffraction and Raman spectroscopy methods in conjunction with the variable angle spectroscopic ellipsometry. To assess the plasmonic properties of TiN thin films, we fabricated periodic nanohole arrays using electron beam lithography (EBL) and reactive ion etching for refractometric sensing.

## 2 Experimental Section

### 2.1 TiN Thin Film Deposition

TiN thin films were deposited on Si (100) wafers and soda-lime glass substrates by reactive RF magnetron sputtering (NANOVAK, NVSP-400) from a 99.995% pure Ti target (Kurt J. Lesker) in a Class 1000 cleanroom environment. The base pressure in the chamber was ∼5 × 10^–6^ Torr. Reactive DC magnetron sputtering with a Zr target (Grade 702, Kurt J. Lesker) was used for chamber cleaning before all TiN depositions. The cleaning was performed with 18 sccm argon gas at different cleaning times (Table S1). Ar/N_2_ gas mixtures were introduced into the system under varying Ar/N_2_ gas flow rates at 200 W (Table S2) for the reactive RF magnetron sputtering of the Ti target. Active heating was used during the process of DC and RF magnetron sputtering at 400 °C, and the deposition rate of films ranged between 2.70 and 4 nm/min.

### 2.2 TiN Nanohole Array Fabrication

**Figure 1:**
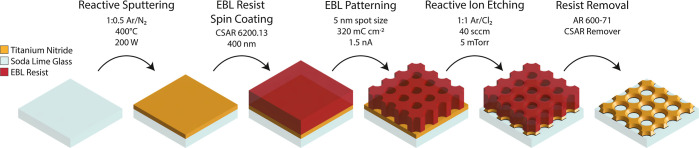
Schematic nanofabrication process flow.

Nanohole array patterning of the thin films was achieved using EBL (Vistec EBPG 5000+). TiN thin films sputtered on a soda-lime glass substrate were initially coated at 500 rpm for 5 s and 5000 rpm at 45 s with CSAR 6200.13 coating and later soft baked for 2 min at 150 °C to yield a baked resist layer with 400 nm thickness. The resist layer was nanopatterned using EBL with an electron beam with a 5 nm spot size, 320 mC cm^–2^ dose, and 1.5 nA current. The remaining structures of the CSAR layer were developed by consecutive treatment with a developer, stopper, deionized water, and 2-propanol, followed by a nitrogen stream dry and 1 min hard-baked at 130 °C. The TiN layer of the developed sample was etched for 60 s with inductively coupled plasma reactive ion etching (Oxford Plasmalab System 100) at 1000 W ICP power, 85 W RF power, a Cl_2_ flow of 20 sccm, an Ar flow of 20 sccm, a process pressure of 5 mTorr, and a substrate temperature of 10 °C. The residue CSAR resist layer was removed from the patterned TiN layer by dipping in CSAR remover solvent (AR 600–71) for 5 min static and 2 min with sonication to remove any remaining resist, followed by nitrogen stream dry. The schematic flow depicted in Figure 1 provides a visual representation of the process, which covers the sequential steps involved in manufacturing TiN thin films, resist coating, patterning using EBL, and etching, as mentioned above.

### 2.3 Characterization

**Figure 2:**
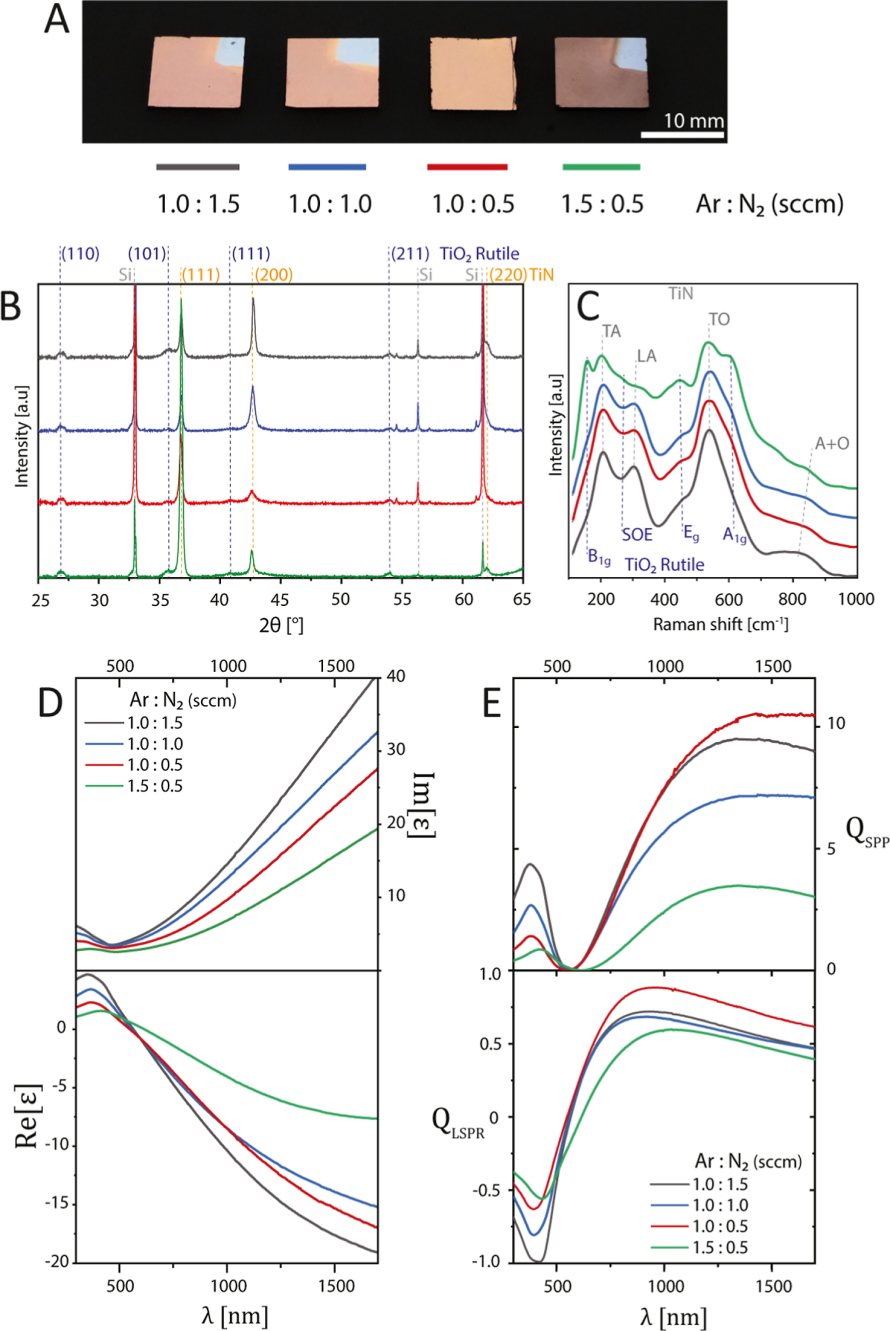
Characterization of optimized TiN thin films for plasmonic applications. (A) Photograph of TiN thin films on 10 × 10 mm Si (100) substrates grown by reactive sputtering under varying Ar/N_2_ flow rates. (B) X-ray diffraction spectra of TiN thin films on Si (100) substrates. See Figures S4 and S5 for more details. The diffraction planes of TiN and rutile TiO_2_ were represented with gray and purple colors, respectively. (C) Raman spectra of TiN on Si (100) substrates. The phonon bands of TiN are annotated in gray and above the stack spectra as follows: TA: transverse acoustic, LA: longitudinal acoustic, TO: transverse optical, A: acoustic, and O: optical. The phonon bands of rutile TiO_2_ are annotated in navy blue and below the stack spectra as following A_1g_, B_1g_, B_2g_, E_g_, and SOE: second order effect due to the interaction of A_1g_ and B_1g_ phonon modes. (D) Real and imaginary parts of the dielectric constant (ε_*r*_ = ε_1_ + *i*ε_2_) from VASE measurements of TiN thin films on Si (100). (E) Calculated plasmonic quality factors for surface plasmon polariton (*Q*_SPP_, ε_1_^2^/ε_2_) and localized surface plasmon resonance (*Q*_LSPR_, |ε_1_|/ε_2_) are from VASE results of TiN thin films on Si (100) substrates.

Optical properties of the deposited thin films were characterized by a variable angle spectroscopic ellipsometer (J.A. Woollam, VASE) system that acquired data at 65, 70, and 75° incident angles. Spectroscopic ellipsometry data was modeled and fitted using WVASE32 software to find real and imaginary parts of the dielectric function. Drude and Lorentz’s models were fitted within the 400–1700 nm wavelength range to analyze the dielectric coefficients. The plasmonic TiN thin films shown in Figure 2 were modeled by using the TiN Drude model, readily available in the WVASE32 software library. The obtained mean squared error (MSE) values were below 0.5 for these samples.

Given that the MSE is below 1.0, no additional roughness has been incorporated into the model. This is because the model fits well with the experimental outcomes. In the presence of the inherent oxide layer on the silicon substrate, a 20 nm native oxide layer was added to the model for all samples. Overall, the film thickness was adjusted to about 165 nm. The fits were applied on a model starting with a silicon substrate, a native oxide, and a metal TiN layer (Drude model) because of the intraband transition of TiN. Film thicknesses were measured with a KLA profilometer, and an ellipsometer was used for thickness control and to conduct the spectroscopic scan. Transmittance measurements were also performed by VASE between 400 and 1700 nm wavelength range. The sheet resistance of the films was measured via a 4-point probe (Cascade Microtech CP4) instrument. The deposited material compositions were analyzed via scanning electron microscopy (SEM)–energy-dispersive X-ray analysis (EDX) (Zeiss, Leo Supra VP 35) mapping for titanium, nitrogen, and oxygen. XRD (Bruker, D8 ADVANCE) measurements were taken between 20° < 2θ < 80° angle range with 0.02° increments providing further compositional and crystallographic information. In addition, Raman (Renishaw inVia Reflex Raman Spectrometer) measurements with a 532 nm incident laser beam were taken between 100 and 1000 cm^–1^. Raman shift range, with 10 mW laser power and 20 s exposure time, to identify the phases of deposited TiN. The thickness of the deposited films was determined using a profilometer (KLA Tencor Surface Profiler). AFM (NanoMagnetic Instruments ezAFM) acquired surface topography and roughness measurements with tapping mode. Detailed surface morphology of the nanohole arrays was visualized by SEM (Zeiss, Leo Supra VP 35).

### 2.4 FDTD Simulations

The finite-difference time-domain (FDTD) simulations were performed by using commercial FDTD software. The excitation light was selected as 400–1700 nm broadband light with a polarization in the *x*-axis. The electric-field intensity distributions were recorded using a 5 nm uniform mesh on an *x*–*z* plane cross-section of nanoholes, 1 nm above the TiN/air interface and 1 nm below the TiN/glass interface. The conformal variant 1 method was used for mesh refinement. In order to ensure metal/dielectric layer precision, an additional custom mesh with a resolution of 1 nm was added to each interface. The boundary conditions were antisymmetric on the *x*-axis, symmetric on the *y*-axis, and steep-angle perfectly matched layers on the *z*-axis in order to reduce the simulation duration. The simulation duration was selected as 500 fs and the auto-shotoff level was set to 10^–7^.

### 2.5 Microspectroscopy

The reflection and transmission spectra of TiN plasmonic square nanohole arrays were measured by using a custom microspectroscopy setup. A fiber-coupled tungsten halogen lamp (Ocean Optics, HL-2000-HP) was utilized as the light source and collimated into the illumination arm of the microscope. The collimated illumination later focused on the back focal plane of the 4× achromat objective (Olympus, PLN 4×) by using an achromatic doublet (Thorlabs, AC254-100-AB) in a cage system. A motorized XYZ stage (Sutter Instruments, MP-285) spatially manipulated the sample. The imaging and spectroscopy arms of the microscope were separated using two broadband nonpolarizing 50:50 beam splitters (Thorlabs, BS-013). The imaging arm was focused on a monochromatic CMOS camera (Basler, model acA4112–30um) using an achromatic doublet (Thorlabs AC254-200-AB). The spectroscopy arm was focused on the 10 μm slit of an astigmatism-free Schmidt-Czerny-Turner imaging spectrometer (Princeton Instruments, IsoPlane SCT 320) with a focal length of 320 mm and 150 lines·mm^–1^ again using an achromatic doublet (Thorlabs AC254-200-AB). The spectral imaging was performed using a back-illuminated EMCCD scientific camera (Princeton Instruments, ProEM HS:1023BX3) coupled to the imaging spectrometer. Wavelength and intensity calibration of the microspectroscopy system was performed using Hg and Ar/Ne lamps (Princeton Instruments IntelliCal) and a NIST traceable LED-based intensity calibration light source (Princeton Instruments IntelliCal), respectively.

## 3 Results and Discussion

The stoichiometric composition and optical properties of TiN thin films strongly depend on the depositing method in use and its conditions. We studied various sputter deposition conditions, including oxygen scavenging with Zr sputtering at different times and different flow rate ratios of Ar/N_2_ to maximize the plasmonic properties of TiN thin films. During deposition, residual oxygen for sputter growth causes impurity because titanium bonds with oxygen rather than nitrogen. Residual oxygen in the deposition chamber often causes unwanted impurities, diminishing the plasmonic performance of TiN thin films in the reactive RF sputtering method. To remedy this issue, an initial session of DC reactive sputter of Zr was performed with an Ar flow rate of 18 sccm at different cleaning/deposition times to minimize oxygen contamination within the chamber before the TiN deposition (see Table S1). After chamber cleaning, the Ar/N_2_ gas ratio was held at 2.0:1.0 sccm, and the RF reactive magnetron sputtering of TiN thin films was performed. The deposited TiN thin films were evaluated by using a VASE (Figure S1a,b). The magnitudes of the real part (ε_1_) and imaginary part (ε_2_) of the dielectric function increased and showed a more metallic profile as we increased the cleaning time. EDX (Figure S1c) shows that increasing the duration of the cleaning step significantly decreased the oxygen impurity in the films, consequently increasing the nitrogen percentage of the fabricated films. Notably, the peak at 0.25 keV is related to the carbon element. However, we did not observe significant changes between 180 and 360 min and concluded that an oxygen scavenging with a duration of 180 min would be sufficient for the later processes.

Various Ar/N_2_ gas ratios were then studied with a constant sputtering time and temperature (process parameters are indicated in Table S2 in SI). By changing the Ar/N_2_ gas flow rates in the reactive RF magnetron sputtering, a range of goldish-colored to brown to black TiN thin films were deposited on Si (100) substrates at 400 °C (Figure S2a and selected thin films in Figure 2a). The TiN thin films with these parameters are also analyzed using a VASE (Figure S2b).

After the initial ellipsometric screening of the plasmonic TiN thin films, the structural characteristics of these films were investigated by using high-resolution X-ray diffraction and Raman spectroscopy. The (004) plane diffraction of the Si substrate was utilized for the angular alignment of the diffraction profiles. In Figure 2B, we observed subtle and broad peaks of rutile TiO_2_ at diffraction angles of ∼26.80° [rutile (110)], ∼35.83° [rutile (101)], ∼40.86° [rutile (111)], and ∼54.57° [rutile (211)]. We also observed A_1g_, B_1g_, B_2g_, E_g_, and SOE phonons of rutile TiO_2_ in the TiN thin films in Figure 2C. Even though these findings showed that we did not completely scavenge all of the oxygen content, its effect on the plasmonic nature of TiN thin films still provides invaluable insights into the effects of oxygen content on the optical properties of TiN. Especially, high Ar/N_2_ (1.5:0.5) flow rates resulted in prominent rutile growth with a highly identifiable B_1g_ phonon at a Raman shift of 158 cm^–1^, a prominent shoulder of A_1g_ phonon at a Raman shift of 604 cm^–1^, and shallow yet identifiable E_g_ phonon at a Raman shift of 446 cm^–1^. Although we were not able to identify rutile formation in our initial XRD screening with a benchtop instrumentation (Bruker, D2 PHASER, scan increment of 0.02° and step 1s), Raman spectroscopy showed great promise to identify low-crystalline impurity formation in the TiN thin films.

The characteristic diffractions of (110), (200), and (220) were observed in XRD angular spectra at 37, ∼43, and 62°, respectively, for all TiN thin films. Although the (220) reflection of TiN was slightly shadowed by the forbidden reflection of Si at the angle of ∼61.67°, it was still identifiable in the analysis. The ratio of (110) and (200) reflections varied as the Ar/N_2_ ratio changed, as shown in Figure 2C. Initially, we observed highly plasmonic thin films with a large magnitude of ε_1_ at a low Ar/N_2_ ratio of 1.0:1.5 sccm. As we increased the Ar flow rate with respect to the N_2_ flow rate, we observed a slight decrease in the magnitude of ε_1_ and more interestingly a decrease in the magnitude of ε_2_. This finding was quite crucial for the plasmonic response of TiN thin films. The quality factor of a plasmonic application can be evaluated using figures of merit such as *Q*_SPP_ and *Q*_LSPR_ accordingly. The *Q*_SPP_ factor is used to assess the propagation length of the surface plasmon polariton (SPP), and the *Q*_LSPR_ factor is used to assess the field enhancement and refractometric sensitivity of the localized surface plasmon resonance (LSPR) at the metal/dielectric interface. In this particular case, as we increase the Ar flow rate, the plasmonic figures of merit parameters, both *Q*_SPP_ and *Q*_LSPR_ at a flow rate ratio of 1.0:0.5, surpassed the plasmonic quality factors of the TiN thin film at a flow rate ratio of 1.0:1.5. The plasmonic quality factors later plummeted at a higher Ar/N_2_ value of 1.5:0.5. This result showed that there is a trade-off between the ratio of Ar/N_2_ flow rates. However, the relationship between the Ar/N_2_ flow rate and plasmonic properties of TiN thin films also depends on the oxygen gas impurity in the system. As the Ar flow increased, more Ti atoms were sputtered and susceptible to reacting with remnant oxygen species in the deposition chamber rather than insufficiently supplied N_2_ gas. The optimized plasmonic response of TiN thin films strongly relies on the deposition chamber vacuum level and oxygen contamination within the chamber. We strongly recommend rigorously optimizing these conditions before the investigation of plasmonic TiN thin films, as they vary from one deposition system to another and should be treated on a case-by-case basis. Hence, it is crucial to establish a correlation between fractured titanium (Ti) and the subsequent release of nitrogen gas (N_2_) into the surrounding environment.

We also investigated the stoichiometry of the plasmonic TiN thin films produced under varying flow rate ratios of Ar/N_2_ using energy-dispersive X-ray spectroscopy in Figures S1 and S3. The optimized flow rate ratio of 1.0:0.5 showed an atomic oxygen percentage below 4%, while the flow ratio of 1.5:0.5 remained above 10%, as shown in Table S3.

In order to assess the plasmonic performance of TiN thin films, we utilized the periodic nanohole array architecture. The nanohole arrays have seen considerable interest in biosensing and energy applications, as they show a combination of SPP and LSPR phenomena. Periodic nanohole arrays mainly allow extraordinary optical transmission (EOT) through subwavelength metal or metal-like thin film perforations at resonant wavelengths.^22^ The EOT system’s optical characteristics depend on the nanohole array’s geometric parameters and dielectric behavior of both thin film and interfacing media. Therefore, the properties of EOT devices can be engineered by varying the design of the system. The resonance frequency of the square nanohole array EOT surface is governed by the following eq 1:^4^
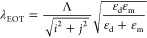
1where Λ represents the period, ε represents dielectric constants of metallic and dielectric media, and *i* and *j* represent diffraction orders. Shabani et al. present a relation between resonance wavelength and properties of TiN EOT devices through FDTD simulations.^23^ They consider different geometrical and material properties and compare these cases by following the shift in the transmission peak; thus, they report similar results to the above equation.^23^ The performance of the TiN-based devices is highly dependent on the fabrication process. The first factor is the high-quality TiN thin film production, and the most common and straightforward method is sputtering.^24,25^ Moreover, studies present other production techniques for high-quality TiN, such as atomic layer deposition (ALD) and molecular beam epitaxy (MBE).^26–28^ The second factor is the fabrication technique for EOT-enabled structures.^29^ Although many studies report techniques for TiN thin film production, the number of studies that report fabricated TiN EOT devices is limited. Wang et al. show the biosensing and perovskite coupling performance of TiN nanoholes produced through selective wet etching of Au–TiN vertically aligned nanotubes VAN structures.^30^ Gherman et al. report that TiN EOTs, fabricated by coating the microsphere lattices, exhibit similar performance to nanohole arrays.^31^ Reese et al. utilized magnetron sputtering and inductively coupled plasma reactive ion etch to fabricate the TiN array.^32^ Although these approaches are novel, the precision of the nanostructures might be problematic. One of the best ways to produce precise nanohole arrays is EBL. Fomra et al. reveal the security application of hexagonal array TiN EOT devices fabricated via EBL and compare the mechanical properties of gold and TiN devices.^33^ Square array EOT devices of sputtered TiN should be investigated.

**Figure 3:**
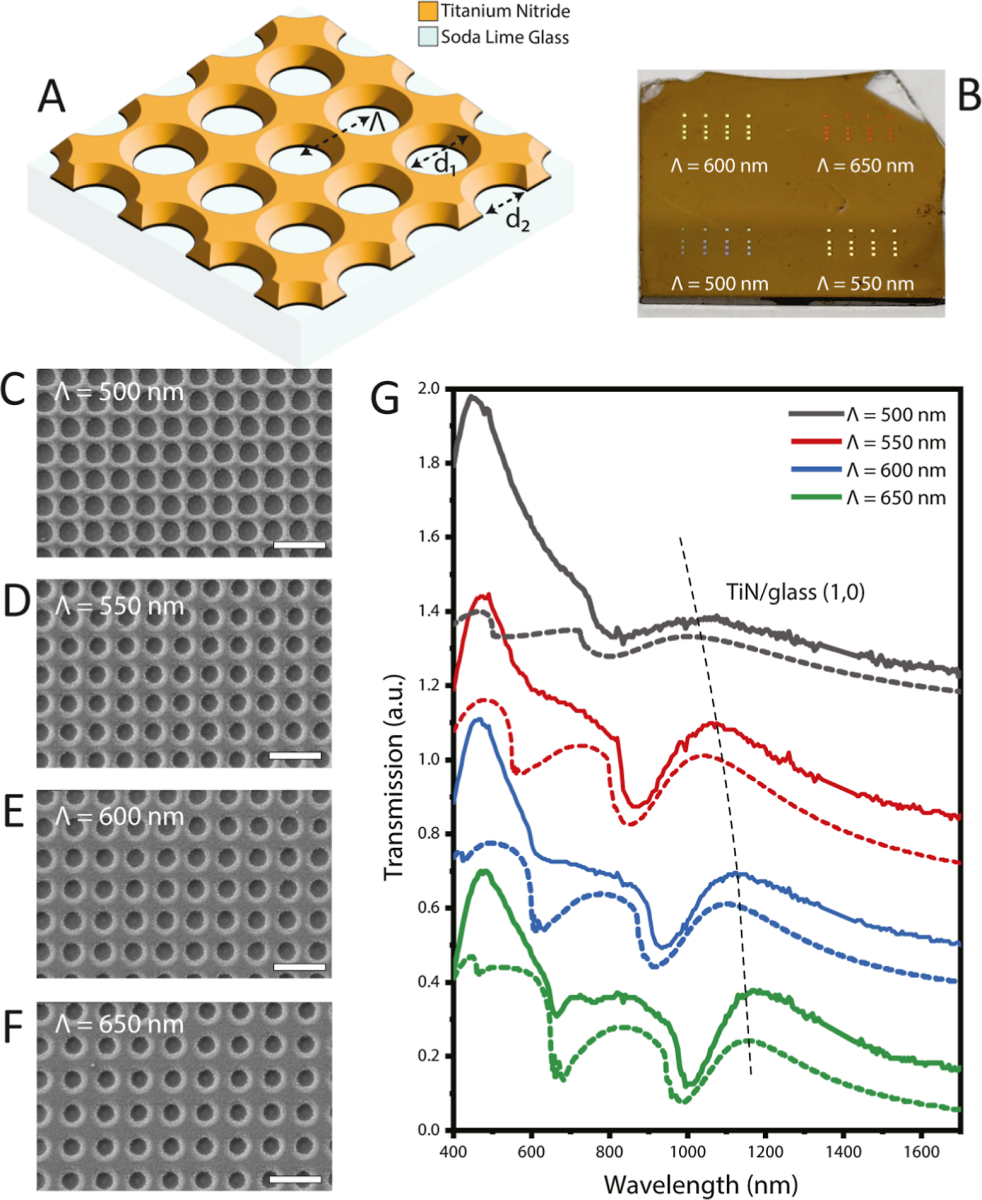
(A) Schematic representation of periodic nanohole arrays on plasmonic TiN thin films. Λ represents the period of the array, and d_1_ and d_2_ represent the top and bottom diameters of the tapered nanohole, respectively. (B) Photograph of the periodic nanohole arrays on plasmonic TiN thin films. The nanohole arrays with various periodicities were fabricated on soda-lime glass substrates with a TiN thin film thickness of 145 nm. Each period cluster contains 16 nanohole arrays with a size of 200 μm × 200 μm. The scanning electron micrographs of nanohole arrays were represented for periods of (C) 500 nm, (D) 550 nm, (E) 600 nm, and (F) 650 nm, where the scale bar represents 1 μm. (G) Transmission spectra of TiN nanohole arrays with each fabricated period represented between wavelengths of 400 and 1700 nm in air. The solid lines represent the experimental measurements and the dashed lines represent the FDTD simulations of the arrays for the corresponding period. The vertical dash line represents the (1,0) SPP at the TiN/glass interface. The SPP modes of TiN/air interfere with higher modes of TiN/glass interface and cannot be observed as a distinct transmission peak.

To demonstrate the EOT phenomenon for plasmonic TiN thin films, periodic TiN nanohole arrays were fabricated from the plasmonic TiN thin film (using Ar/N_2_ flow rate ratio of 1.0:0.5 sccm) samples using EBL as shown in Figure 3. The characteristic TiN transmission peak at 440 nm was visible for all samples. Unlike the thin film, the nanohole arrays showed considerable dips and peaks in transmittance in the spectral range of 600–1300 nm due to the EOT phenomenon, as shown in Figure S7. As hole array periodicity increased, the peaks due to EOT undertook a red shift. This phenomenon can be explained by eq 1 as the wavelength of the excited surface plasmon mode (λsp) depends on the periodic constant of the whole array. In addition, due to the increasing distance between the holes, the LSPR interaction decreased, resulting in lower transmittance peak intensity for larger periodicity. This EOT behavior agrees with the findings of Shabani et al., showing surface plasmons from the oscillation of free electrons at the intermetallic and dielectric interface support EOT with tunable optical behavior.^23^

**Figure 4:**
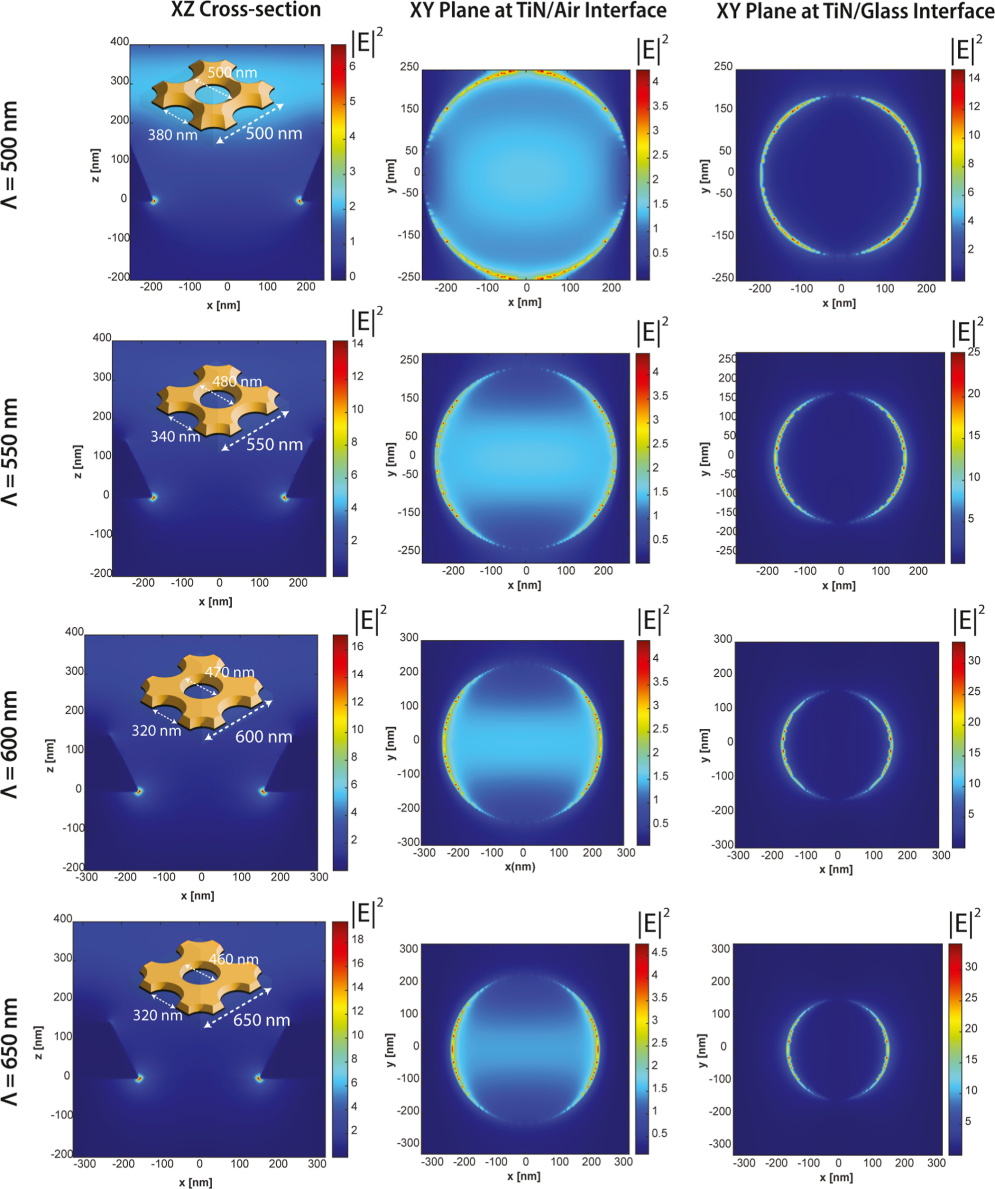
Electric field intensity distributions (|*E*|^2^) in the nanohole cross-sectional plane (x–z), at the TiN/air interface, and at the TiN/glass interface at resonance wavelengths of (1,0) modes were plotted. The geometric parameters of the nanoholes were extracted from the SEM micrographs in Figure 3.

The sensing performance of EOT devices is affected by the physical features of the metal surface since plasmons propagate at the metal–dielectric interface. Therefore, controlling the surface morphology and roughness is crucial for high EOT sensitivity. Rough surfaces and edges cause additional scattering and optical loss, which results in a lower sensing performance. AFM image of TiN film used for EOT production is represented in Supporting Information. The root mean square (RMS) of thin TiN films was equal to 3.13 nm, as shown in Figure S6. This value is acceptable compared with previously reported RMS values of sputtered TiN films.^34–36^ The image of the TiN nanohole array device is presented in Figure 3B, which clearly shows the locations of the nanohole arrays due to the coupling of visible light. Figure 3 shows a photograph and SEM images of plasmonic TiN nanohole arrays with periodicities of 500, 550, 600, and 650 nm. The top apertures of the nanoholes were not directionally etched due to the anisotropic etching characteristics of Cl_2_ gas. The top and bottom diameters of the nanohole apertures are represented in Figure 3A as we show their transmission and reflection spectra. Transmission measurements were initially performed in the air to investigate surface plasmons’ behavior as they propagate over the contact between the TiN and dielectric interface. The transmission spectra of TiN nanohole arrays, with each fabrication period, are represented throughout the wavelength range of 400–1700 nm in Figure 3G. Solid lines depict the experimental observations, while the FDTD simulations of the arrays for the equivalent periods are represented by dashed lines. FDTD simulations indicated that the (1,0) SPP mode was observed at the interface between a TiN nanohole array and glass within the 600–1300 nm wavelength range in air. The detailed electric field distributions in the cross-sectional plane, TiN/air, and TiN/glass interfaces are shown at the corresponding resonance wavelength in Figure 4. According to Figure 3G, this result also coincided with experimental measurements (solid lines) for the transmission spectra of the TiN nanohole arrays. The transmission peak at 440 nm, a distinctive feature of TiN, was observed in all samples. Furthermore, it can be observed from both the FDTD simulation and experimental graphs that there is a rightward shift in the wavelength range from 1000 to 1200 nm when the period increases from 500 to 650 for the (1,0) SPP at the TiN/glass interface.

**Figure 5:**
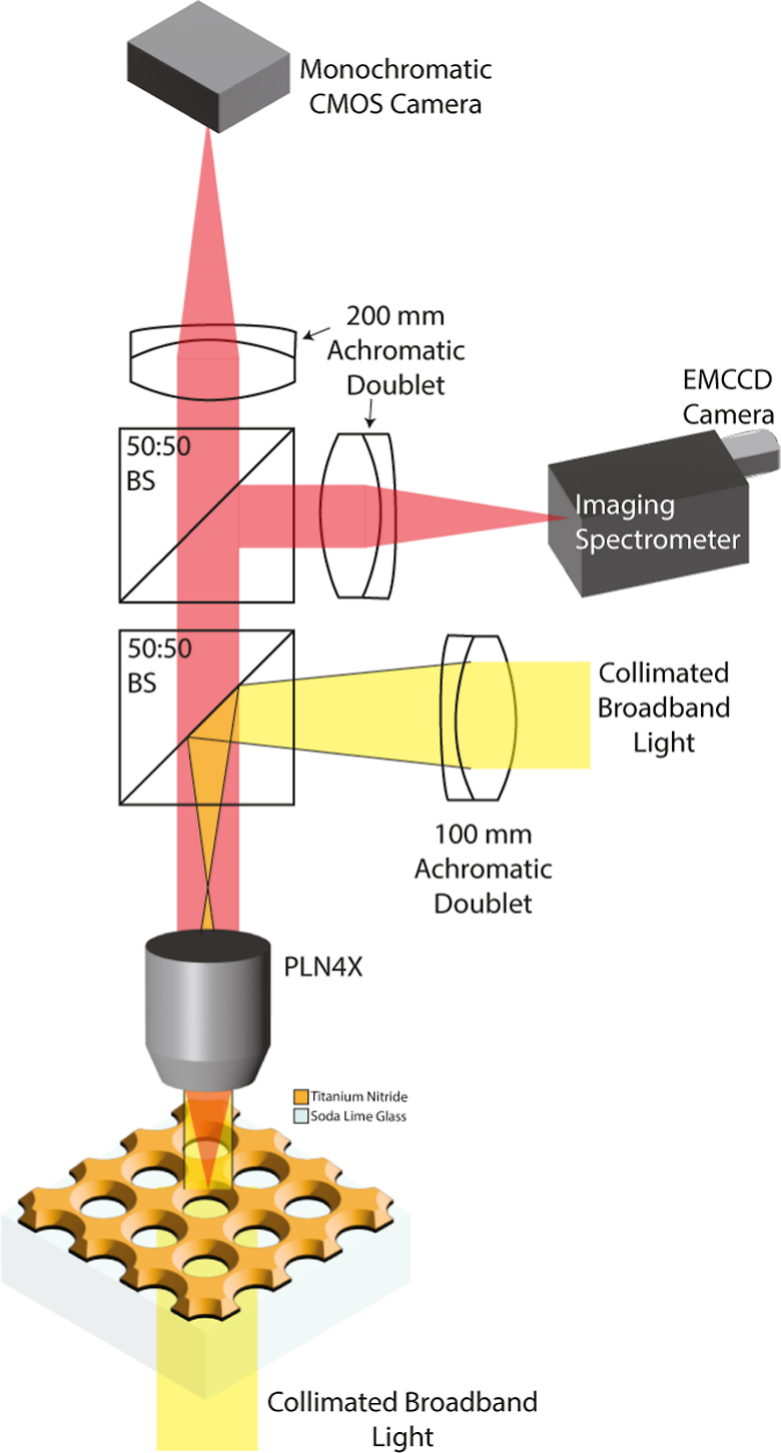
Schematic representation of the custom reflection/transmission microspectroscopy setup. In the illumination arm (yellow), the collimated broadband light is focused onto the back focal point of the achromatic microscope objective to obtain plane-wave illumination on the nanohole array sample. The imaging/spectroscopy arm was split using nonpolarizing 50:50 beam splitters. The collimated broadband light below the sample stage was turned off for reflection measurements. Reciprocally, the light source focused on the back focal point of the microscope objective was turned off for transmission measurements. Achromatic doublets with broadband antireflective coatings (400–1100 nm) were used in the custom microspectroscopy setup to ensure spectral purity.

In order to correctly excite the SPP and induce the EOT phenomenon, we utilized a custom microspectroscopy setup where we ensured plane-wave illumination from both the top and the bottom, as shown in Figure 5. The arrays were visualized using the imaging arm of the microscope using a monochromatic CMOS camera and spectrally resolved using an EMCCD camera coupled imaging spectrometer.

**Figure 6:**
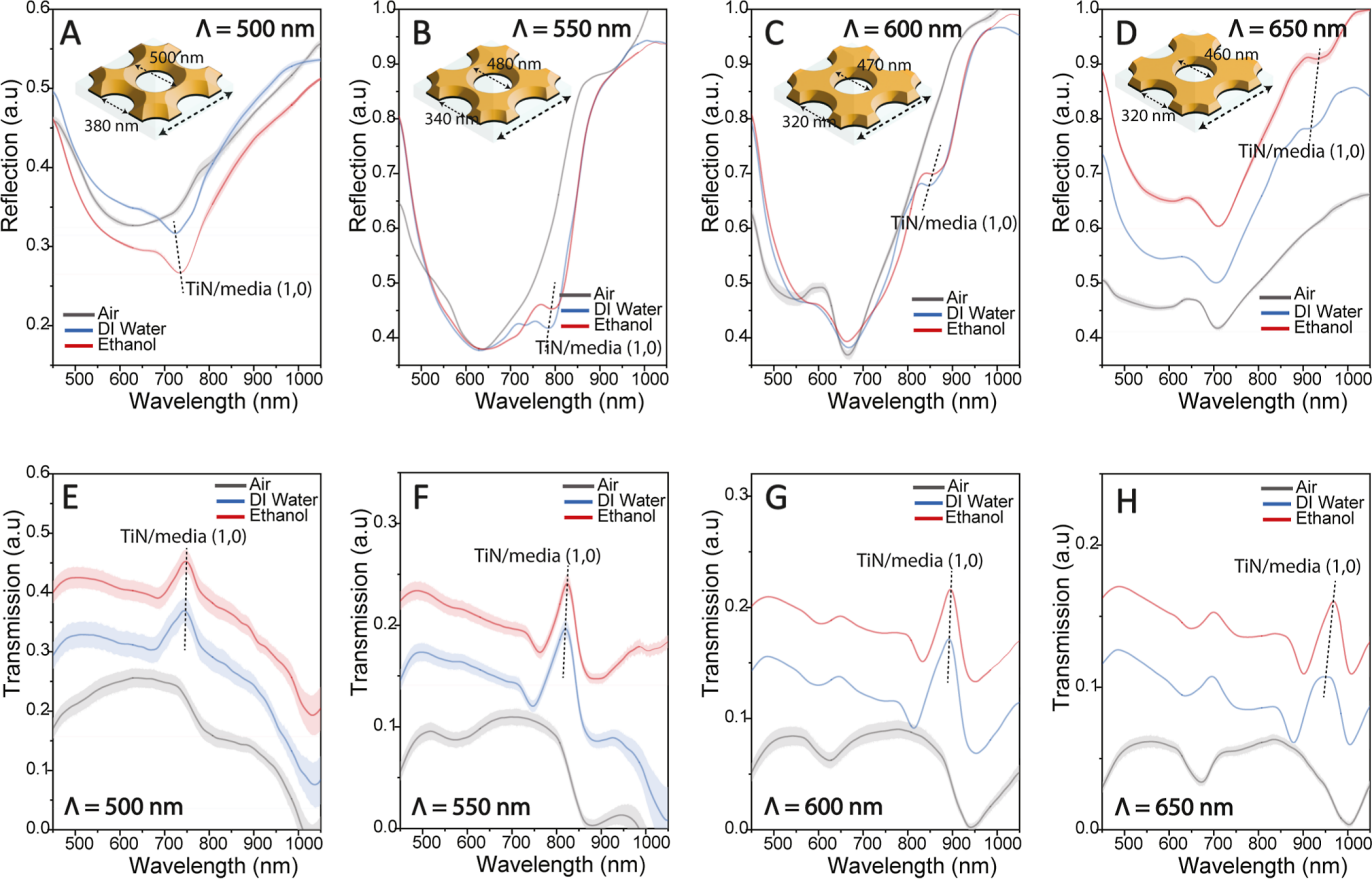
Reflection spectra of TiN nanohole arrays with nanohole periods of (A) 500 nm, (B) 550 nm, (C) 600 nm, and (D) 650 nm in the air (*n* = 1.0003), deionized water (*n* = 1.33459), and ethanol (*n* = 1.3614). The schematic representation of the tapered nanohole structure is illustrated as insets in each subfigure with the top and bottom diameters of the nanoholes. The transmission spectra of TiN nanohole arrays with nanohole periods of (E) 500 nm, (F) 550 nm, (G) 600 nm, and (H) 650 nm were plotted in the air (*n* = 1.0003), deionized water (*n* = 1.33459), and ethanol (*n* = 1.3614). The slightly inclined vertical dashed lines in all figures represent the (1,0) SPP mode at the TiN/media interface. The shaded area of each line represents the error bars of spectral measurements over 16 identically fabricated 200 μm × 200 μm nanohole arrays.

The refractometric sensing capabilities of the TiN nanohole array were evaluated by manipulating the surrounding medium’s refractive index. The selection of the refractive index range was made within the values of 1.0 (air), 1.33 (DI water), and 1.36 (ethanol) to track the (1,0) TiN/media modes, shown in Figure 6. The spectral reflectance measurements showed a shallow dip where the resonance conditions met in eq 1. The reflection dip was observed at the 700–800 nm band for the nanohole period of 500 nm and successively red-shifted as the period of the nanohole array increased. Especially, the reflection dip was hard to identify for periods of 600 and 650 nm. On the other hand, spectral transmission measurements showed a prominent EOT peak for all periods. Variations in the refractive index of the medium situated in the evanescent field induce alterations in the polarizability, subsequently causing shifts in the peak of the EOT. For the EOT, the plasmonic resonance wavelength was observed at 700–800 nm for the period of 500 nm. As in the reflectance and transmission spectrum, a shift to the right was observed as the period increased. Figure 6A–D illustrates an almost invisible but noticeable displacement of the reflection dip (the TiN/media (1,0) mode) when comparing DI water and ethanol. The plasmonic resonance shifts in reflection from DI water to ethanol were calculated to be 12.85 nm for 500 periods and 20.06 nm for 650 periods, respectively. Furthermore, Figure 6E–H exhibits a tiny displacement (shown by dashed lines) except for the scenario with a periodicity of 500 nm. The resonance shift in the transmittance graph between DI water and ethanol was determined to be 3.16 nm for 500 periods and 17.69 nm for 650 periods, respectively. Despite the small magnitude of the plasmonic resonance wavelength shift for transmittance, it is nevertheless detectable. As demonstrated in Figure 6A–D, the nanohole structures exhibit significant variations between their upper and lower diameters due to the limited achievable of high anisotropy in reactive ion etching. This difference is comparatively less in the 650 s, as evidenced by SEM images depicted in Figure 3C,F, thus indicating that it is detectable specifically in the period of 650 s.

The primary sensitivity parameter in refractometric sensing, known as the bulk sensitivity (S_B_), measures the capacity to detect small variations in the refractive index of the dielectric medium in direct contact with the sensor’s surface. The calculation of bulk sensitivity involves determining the response (Δλ) per refractive index variation (Δ*n*), which is then expressed in refractive index units (RIUs).^4^ The change in resonance wavelength per unit refractive index change of the medium was calculated using linear fitting analysis, and the slope of the Δλ vs Δ*n* plot measures the *S*_B_.^37^ The refractive index sensitivity of plasmonic TiN periodic nanohole arrays was measured as ∼180 nm/RIU. Although it is not on par with Au nanohole arrays shown in Table S4, TiN can still be improved for better and maybe for a unique application where we can exploit the thermal and mechanical properties of TiN. The sensitivity of a system is inherently linked to the refractive index of the medium. Consequently, efforts to enhance sensitivity can be implemented through modifications to either the medium or the nanostructure of the TiN.

## 4 Conclusions

This study presents highly plasmonic TiN thin films and nanostructures using a low-power and bias-free reactive sputtering process. We have investigated the optical and structural properties of the TiN films and their dependence on several processing parameters, such as substrate material, reactive gas flow ratio, and film thickness. Then, we have also fabricated periodic square nanohole arrays of TiN and validated that strong localized plasmonic resonances are supported. The sensitivity of the refractive index of plasmonic TiN nanohole arrays was determined to be approximately 180 nm/RIU. This study offers significant contributions to understanding the behaviors exhibited by TiN nanohole arrays in the context of refractometric sensing applications. It also highlights the abundant availability of TiN and its efficient fabrication process, making it highly suitable for large-scale production, and the full range of possibilities and advantages associated with using refractory metal nitrides in plasmonics with existing CMOS process technologies to enable more practical applications.

## Supporting Information Available

Optimization of Zr cleaning time for plasmonic TiN thin films. Optimization of Ar: N_2_ gas flow ratio for plasmonic TiN thin films. Investigation of plasmonic TiN thin film stoichiometry. Crystallographic investigation of plasmonic TiN thin films. Surface Morphology of plasmonic TiN thin film using in nanohole fabrication. Transmittance spectra of plasmonic TiN nanohole arrays and unpatterned thin film. Literature Comparison. (PDF)
