# Supplementary material for: Plasmonic Titanium Nitride Nanohole Arrays for Refractometric Sensing
Source: ACS Appl Nano Mater. 2023 Nov 14;6(22):20612–22. doi: 10.1021/acsanm.3c03050 (PMC10684111; doi:10.1021/acsanm.3c03050)
Supplement: Supplementary file 1 — an3c03050_si_001.pdf [file an3c03050_si_001.pdf]

Supporting Information

Plasmonic Titanium Nitride Nanohole Arrays for Refractometric Sensing

Beyza Nur Günaydın[1,2], Mert Gülmez[1+], Milad Torabfam[1,2+], Zeki Semih Pehlivan[1,2,3], Atacan Tütüncüoğlu[1,2], Cemre Irmak Kayalan[1,2], Erhan Saatçioğlu[4], Mustafa Kemal Bayazıt[2], Meral Yüce[2,6*], Hasan Kurt[4,5,6*]

[1] Faculty of Engineering and Natural Sciences, Sabanci University, Tuzla, Istanbul, 34956, Turkey

[2] SUNUM Nanotechnology Research and Application Centre, Sabanci University, Tuzla, Istanbul, 34956, Turkey

[3] Department of Materials Science and Metallurgy, University of Cambridge, Cambridge, CB2 3EQ, UK

[4] Research Institute for Health Sciences and Technologies (SABITA), Istanbul Medipol University, Beykoz, Istanbul, 34810, Turkey

[5] School of Engineering and Natural Sciences, Istanbul Medipol University, Beykoz, Istanbul, 34810, Turkey

[6] Department of Bioengineering, Royal School of Mines, Imperial College London, London, SW7 2AZ, UK

[+]Equal contribution

[*]Corresponding Authors: meralyuce@sabanciuniv.edu & h.kurt@imperial.ac.uk

Supporting Information

1. Optimization of Zr cleaning time for plasmonic TiN thin films

Table S1. The list of films fabricated at various sputtering conditions.

No	Zr cleaning time (min.)	N_2 (sccm)	Ar (sccm)	Deposition T (ºC)	Pressure (Torr)	RF power (W)
**1**	30	1	2	400	10^{-6}	200
**2**	60	1	2	400	10^{-6}	200
**3**	180	1	2	400	10^{-6}	200
**4**	360	1	2	400	10^{-6}	200

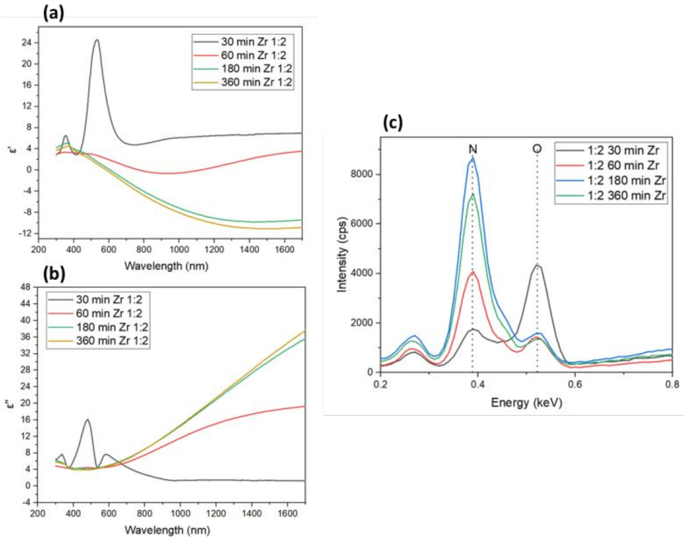

Figure S1. The characterization of TiN thin films for plasmonic applications. **(a)** Real and **(b)** imaginary parts of the dielectric constant ($\varepsilon_r = \varepsilon' + i\varepsilon''$) from variable angle spectroscopic ellipsometry (VASE) measurements of TiN thin films on Si (100). **(c)** SEM-EDX spectra of TiN thin films for different Zr cleaning times at constant Ar:N_2 (2.0:1.0) sccm**.**

2. Optimization of Ar: N_2 gas flow ratio for plasmonic TiN thin films

Table S2. The list of TiN films fabricated at various sputtering conditions (e.g. Zr cleaning time, N_2, and Ar gas flow rate, deposition temperature, base pressure, and RF power)

Sample No	Zr cleaning time (min.)	N_2 (sccm)	Ar (sccm)	Deposition Temp. (ºC)	Base pressure (Torr)	RF power (W)
1	180	2	1	400	10^{-6}	200
2	180	1.5	1	400	10^{-6}	200
3	180	1.7	1	400	10^{-6}	200
4	180	1.3	1	400	10^{-6}	200
5	180	1	1	400	10^{-6}	200
6	180	0.5	1	400	10^{-6}	200
7	180	0.5	1.2	400	10^{-6}	200
8	180	0.5	1.5	400	10^{-6}	200
9	180	0.5	2	400	10^{-6}	200
10	180	0.5	4	400	10^{-6}	200

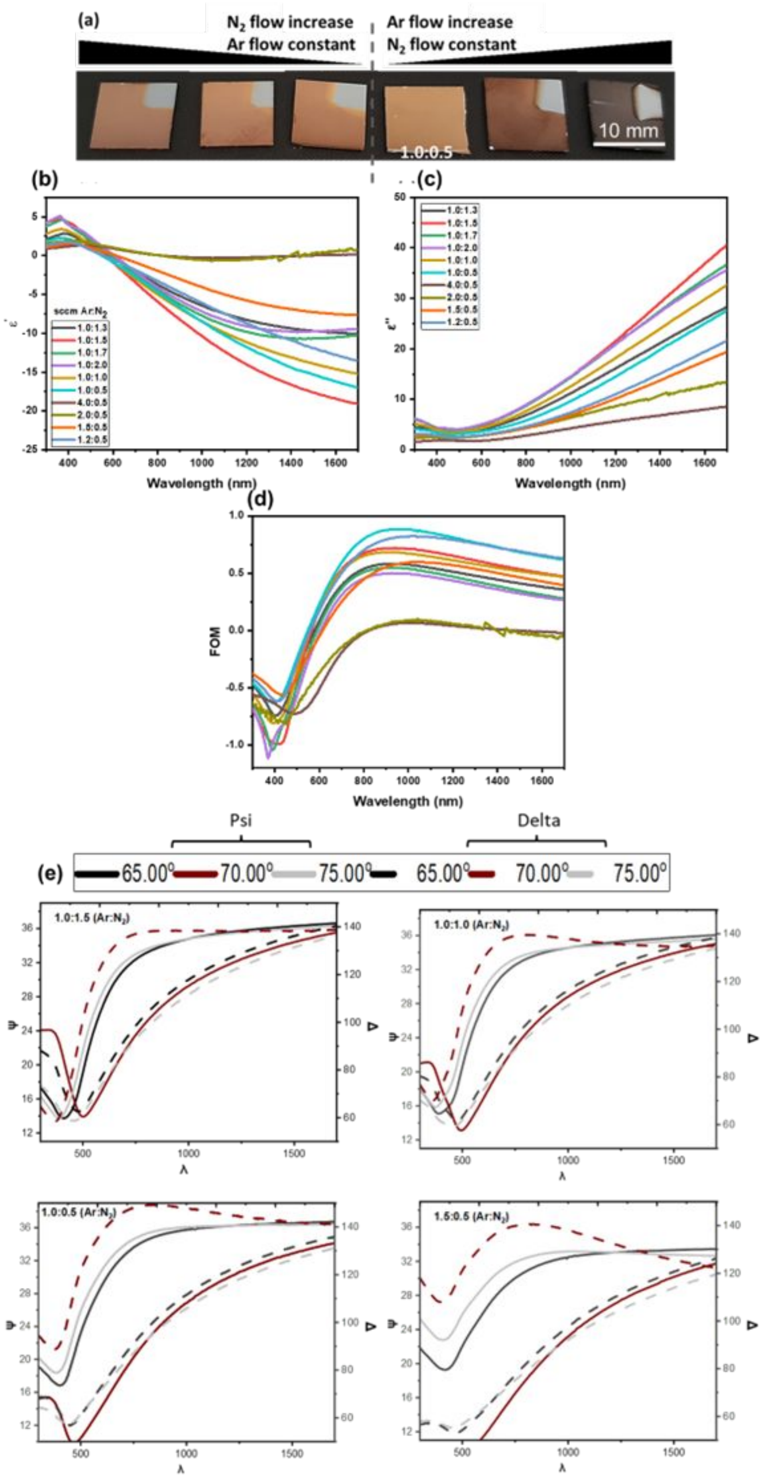

Figure S2. The optical characterization of plasmonic TiN thin films. **(a)** Photograph of TiN thin films on 10 × 10 mm Si (100) substrates grown by reactive sputtering under varying Ar:N2 flow rates. **(b)** Real and **(c)** imaginary parts of the dielectric constant (ε=ε'+iε'') from variable angle spectroscopic ellipsometry (VASE) measurements of TiN thin films on Si (100). **(d)** The calculated localized surface plasmon resonances (FOM=|ε'|/ε'') from VASE results of TiN thin films on Si (100) substrates. **(e)** Spectral ellipsometry analysis to investigate the Psi (ψ) and Delta (Δ) values of selected planar titanium nitride (TiN) thin films fabricated at various Ar:N2 ratios in the wavelength range of 300-1700 nm by J.A Woollam VASE Ellipsometer. The measurements were conducted at incidence angles of 65°, 70°, and 75°.

3. Investigation of plasmonic TiN thin film stoichiometry

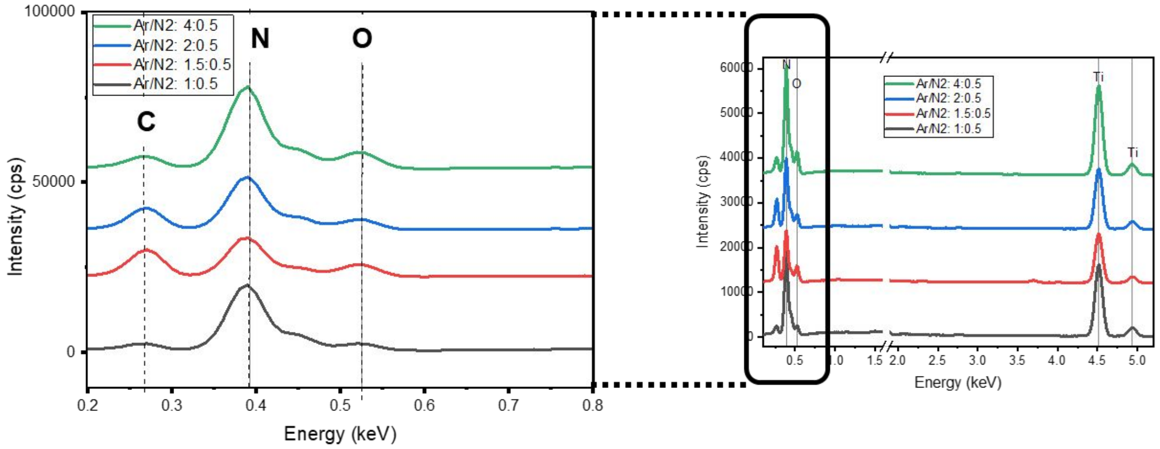

Figure S3. SEM-EDX images of TiN thin films with varying ratios of argon to nitrogen in terms of sccm. The graph on the right displays a scanning range of 0.2 to 5.5 keV, whereas the image on the left focuses on a narrower range of 0.2 to 0.8 keV in order to provide a more detailed observation of the N - O relation.

Table S2. The atomic weight percentage of elements for TiN thin films at different Ar: N_2 ratios (sccm) obtained by SEM-EDX analysis to present the stoichiometry of TiN thin films.

AT%	1.0:0.5	1.5:0.5	2.0:0.5	4.0:0.5
N	32.19	30.77	32.98	35.07
O	3.94	10.92	7.88	9.63
Si	39.63	32.88	32.55	25.94
Ti	24.24	25.42	26.59	29.36
**(N+O)/Ti**	1.49	1.64	1.54	1.52

4. Crystallographic investigation of plasmonic TiN thin films

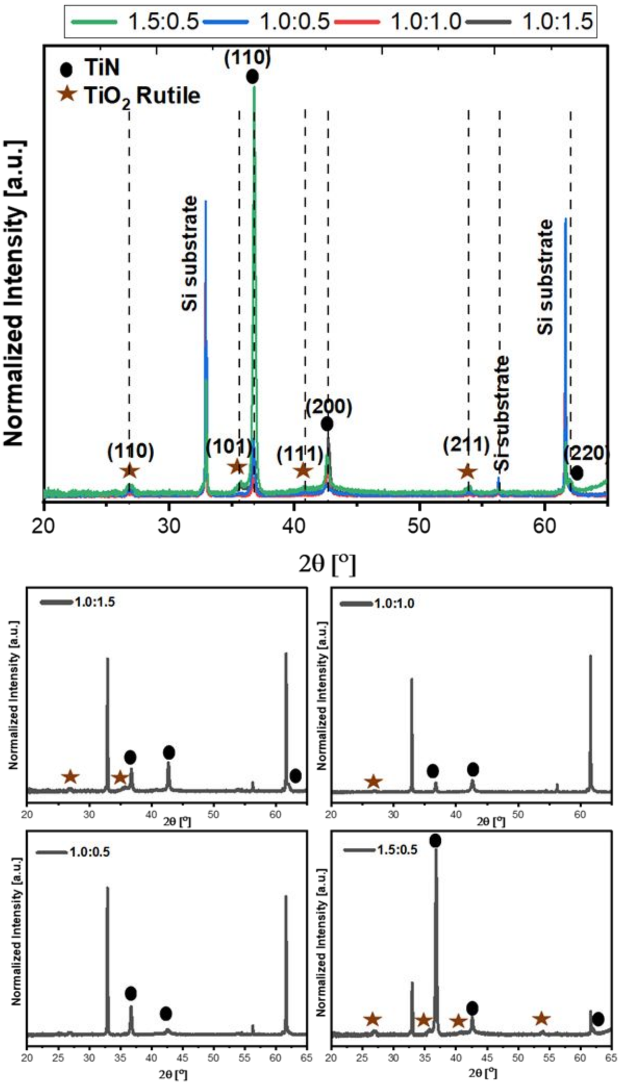

Figure S4. The characterization of TiN thin films for plasmonic applications. The X-ray diffraction spectra of TiN thin films at various Ar:N2 ratios on Si (100) substrates. The diffraction planes of TiN and rutile TiO2 were represented with black dots and stars of brown color, respectively.

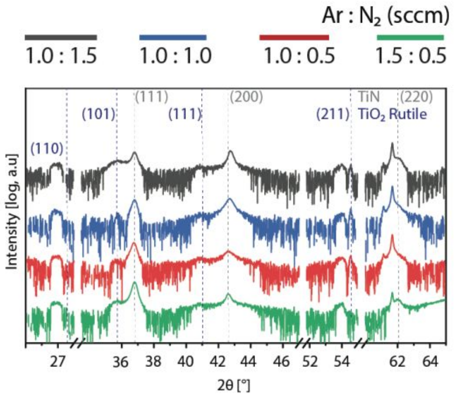

Figure S5. X-ray diffraction spectra of TiN thin films on Si (100) substrates. Intensity counts were represented in a logarithmic scale. The diffraction planes of TiN and rutile TiO_2 were represented with grey and purple colors, respectively.

5. Surface Morphology of plasmonic TiN thin film using in nanohole fabrication

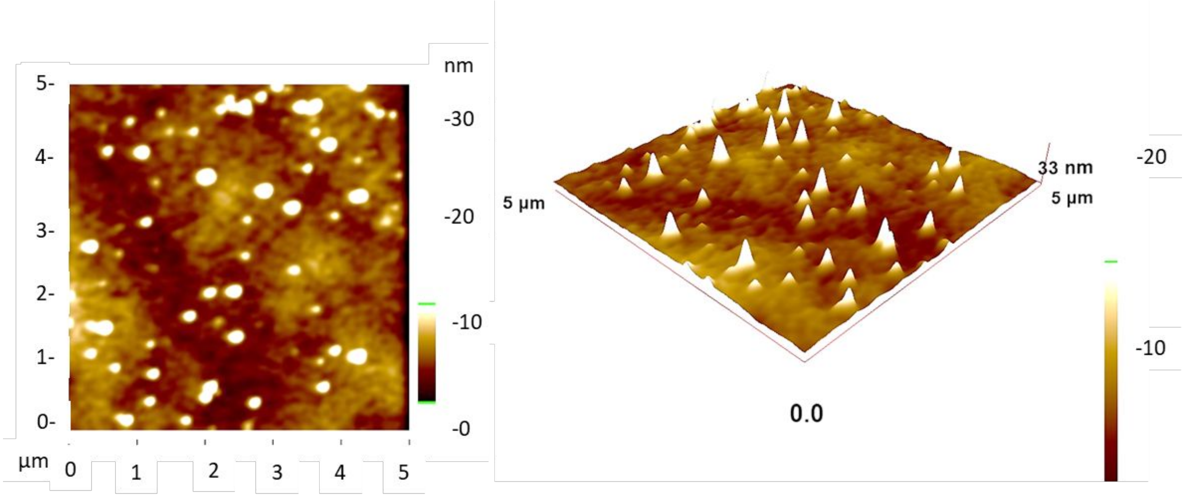

Figure S6. AFM image of plasmonic TiN thin film sputtered under 1.0:0.5 Ar/N_2 flow. 2D (left) and 3D (right) topography images of plasmonic TiN thin film.

6. Transmittance spectra of plasmonic TiN nanohole arrays and unpatterned thin film

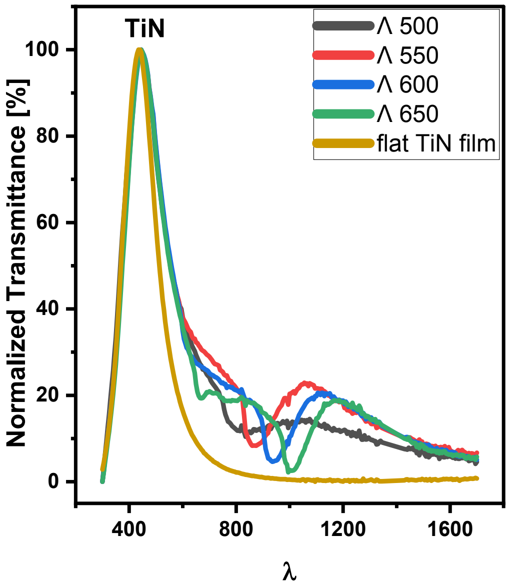

Figure S7. The transmittance spectra of the plasmonic TiN thin film and TiN nanohole arrays with periods ranging from 500 nm to 650 nm in air.

7. Literature Comparison

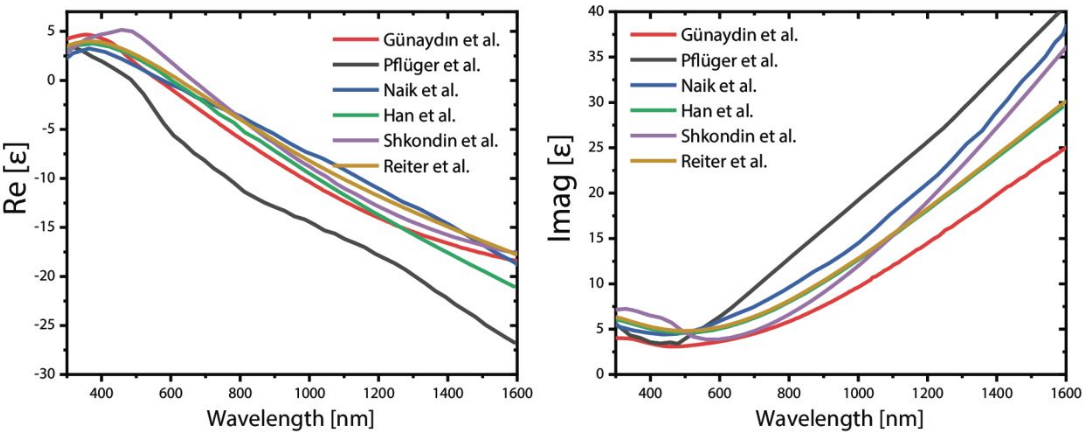

Figure S8. The comparison of dielectric behavior of plasmonic TiN thin films with the earlier works in the literature.[1–5]

Table S3. The list of refractometric sensitivity for the sensitivity performances of various materials.

**Materials**	**Refractive index sensitivity (nm/RIU)**
Gold nano-cross array on a gold nanofilm and a SiO_2 spacer[6]	872
Gold-coated circular nano-film photonic crystal fiber[7]	45003.05
Triple layer MgF_2-gold-MgF_2 coated nano metal films[8]	28,046.205
Gold/silicon complementary grating structure[9]	1642
Gold film coated with multilayer carbon nanotubes and platinum nanoparticles composite (MWCNT/PtNPs)[10]	5923
Gold- and MgF_2-coated nano metal films[11]	27,958.49

Supporting Information

References

(1) Pfliiger, J.; Fink, J.; Weber, W.; Bohnen, K.-P.; Crecelius, G. Dielectric Properties of TiC_x, TiN_x, VC_x, and VN_x from 1.5 to 4.0 eV Determined by Electron-Energy-Loss Spectroscopy. *Phys Rev B* **1984**, *30* (3), 1155–1163. https://doi.org/10.1103/PhysRevB.30.1155.

(2) Naik, G. V; Shalaev, V. M.; Boltasseva, A.; Naik, G. V; Shalaev, V. M.; Boltasseva, A. Alternative Plasmonic Materials: Beyond Gold and Silver. *Advanced Materials* **2013**, *25* (24), 3264–3294. https://doi.org/10.1002/ADMA.201205076.

(3) Han, W.; Reiter, S.; Schlipf, J.; Mai, C.; Spirito, D.; Jose, J.; Wenger, C.; Fischer, I. A. Strongly Enhanced Sensitivities of CMOS Compatible Plasmonic Titanium Nitride Nanohole Arrays for Refractive Index Sensing under Oblique Incidence. *Opt Express* **2023**, *31* (11), 17389. https://doi.org/10.1364/OE.481993.

(4) Shkondin, E.; Repän, T.; Takayama, O.; Lavrinenko, A. V. High Aspect Ratio Titanium Nitride Trench Structures as Plasmonic Biosensor. *Opt Mater Express* **2017**, *7* (11), 4171. https://doi.org/10.1364/OME.7.004171.

(5) Reiter, S.; Han, W.; Mai, C.; Spirito, D.; Jose, J.; Zöllner, M.; Fursenko, O.; Schubert, M. A.; Stemmler, I.; Wenger, C.; Fischer, I. A. Titanium Nitride Plasmonic Nanohole Arrays for CMOS-Compatible Integrated Refractive Index Sensing: Influence of Layer Thickness on Optical Properties. *Plasmonics* **2023**, *18* (3), 831–843. https://doi.org/10.1007/s11468-023-01810-3.

(6) Chen, H.; Wang, X.; Zhang, J.; Rao, X.; Yang, H.; Qi, Y.; Tang, C. Theoretical Study of Surface Plasmonic Refractive Index Sensing Based on Gold Nano-Cross Array and Gold Nanofilm. *Physica B Condens Matter* **2023**, *655*, 414746. https://doi.org/10.1016/J.PHYSB.2023.414746.

(7) Abdullah, H.; Ahmed, K.; Mitu, S. A. Ultrahigh Sensitivity Refractive Index Biosensor Based on Gold Coated Nano-Film Photonic Crystal Fiber. *Results Phys* **2020**, *17*, 103151. https://doi.org/10.1016/J.RINP.2020.103151.

(8) Abdullah, H.; Ahmed, K.; Alam, M. S.; Rashed, A. N. Z.; Mitu, S. A.; Al-Zahrani, F. A.; Anowar Kabir, M. High Sensitivity Refractive Index Sensor Based on Triple Layer MgF2-Gold-MgF2 Coated Nano Metal Films Photonic Crystal Fiber. *Optik (Stuttg)* **2021**, *241*, 166950. https://doi.org/10.1016/J.IJLEO.2021.166950.

(9) Chinese Physics B. **2021**. https://doi.org/10.1088/1674-1056/abd690.

(10) Jiang, X.; Wang, Q. Refractive Index Sensitivity Enhancement of Optical Fiber SPR Sensor Utilizing Layer of MWCNT/PtNPs Composite. *Optical Fiber Technology* **2019**, *51*, 118–124. https://doi.org/10.1016/J.YOFTE.2019.05.007.

(11) Ahmed, K.; Alzain, M. A.; Abdullah, H.; Luo, Y.; Vigneswaran, D.; Faragallah, O. S.; Eid, M. M. A.; Rashed, A. N. Z. Highly Sensitive Twin Resonance Coupling Refractive Index Sensor Based on Gold- and MgF2-Coated Nano Metal Films. *Biosensors 2021, Vol. 11, Page 104* **2021**, *11* (4), 104. https://doi.org/10.3390/BIOS11040104.
